# Grossesse abdominale momifiée

**DOI:** 10.11604/pamj.2016.25.230.10857

**Published:** 2016-12-08

**Authors:** Abdi Ahmed Bonahy, Houssam Sabbah, Ahmed Baba Abdeljelil, Moubarak Mahmoudi

**Affiliations:** 1 Département Mère et Enfant, Faculté de Médicine de Nouakchott, Mauritanie; 2 Maternité du Centre Hospitalier National (CHN) de Nouakchott, Mauritanie

**Keywords:** Lithopedion, grossesse ectopique, grossesse abdominale, Lithopedion, ectopic pregnancy, abdominal pregnancy

## Abstract

Le lithopédion est une grossesse abdominale ancienne arrêté et calcifié. La prise en charge, elle reste non codifiée. En effet, si certains auteurs préconisent un traitement chirurgical, d'autres l'expectative. Nous rapportons une observation concernant une femme de 46 ans se présentant pour une masse pelvienne révélant une grossesse abdominale calcifiée évoluant depuis 8 ans.

## Abstract (en)

Lithopedion is a retained abdominal pregnancy that evolves to fetal death and calcification. Its management remains uncodified. Indeed, some authors recommend surgical treatment, while others recommend to wait. We report the case of a 46-year old woman presenting with a pelvic mass revealing a calcified abdominal pregnancy that had been evolving for 8 years.

## Introduction

Le lithopédion est une grossesse extra utérine ancienne qui a involeuée puis s'est calcifiée au fils du temps. Ce mot vient du grec et il signifie littéralement «enfant de pierre » [1]. Il s'agitd'une forme rare et de diagnosticdifficile nécessitant souvent l'apport de la radiologie. Il représente 1.5 à 2% des grossesses ectopiques [2]. Dans de rare cas, le diagnostic s'est fait lors d'une intervention chirurgicale voir même au cours d'une autopsie [3]. La prise en charge est chirurgicale. Nous rapportons le cas d'une patiente âgée de 46ans, multipare et dont le lithopédion remontait à 8 ans avant son diagnostic.

## Patient et observation

**Figure 1:**
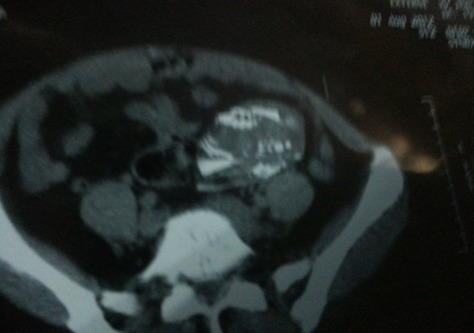
Scanner abdominale, masse calcifiée avec des membres embryonnaires

**Figure 2:**
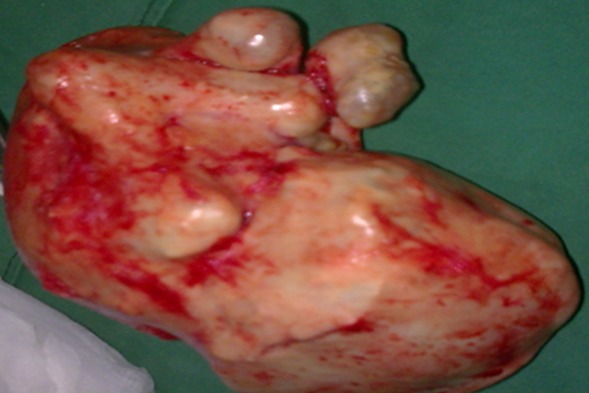
Pièce opératoire (lithopedion)

Il s'agit d'une patiente âgée de 46 ans, sixième geste cinquième pare, ayant cinq enfants bien portants, dont le dernier accouchement remontait à 8 ans. Elle nous a consultée pour des douleurs pelviennes chroniques avec sensation de pesanteur pelvienne. Les antécédents étaient sans particularités et la patiente avait un cycle régulier. Devant la persistance des douleurs pelviennes chronique associées à des épisodes de constipations, la patiente a consulté en gynécologie. L'interrogatoire de la patiente révèle la notion d'une aménorrhée de 5mois avec des signes sympathique et ceci quelques mois avant sa dernière grossesse normale. L'examen clinique semblait normal en dehors d'une douleur à la palpation abdominale. Un bilan radiologique a été demandé. Il s'agissait de l'échographie, qui a mis en évidence une masse sus utérine calcifiée de 37 cm, dont l'origine était difficile à déterminée. Quant auScanner, il a confirmé la présence d'une structure calcifiée par endroit avec une tête et des membres bien individualisés (Figure 1). Le bilan biologique était normal. La décision d'une laparotomie est alors prise. A l'ouverture l'utérus ainsi que les ovaires étaient sains. Par ailleurs, nous avons noté la présence d'une volumineuse masse calcifiée entourée par l'épiploon contenant un embryon momifié (Figure 2). Nous avons effectué l'ablation chirurgicale de la masse ainsi qu'une exérèse d'une portion de l'épiploon.

## Discussion

Le lithopédion est une forme rare de grossesse ectopique. Le premier cas rapporté dans la littérature a été décrit par un chirurgien du nom d'Albucasis au X^ème^ siècle [3]. Cette pathologie est la résultante de plusieurs facteurs dont les principaux sont: l'implantation ectopique de la grossesse, le non diagnostic de cette grossesse qui va évoluée jusqu'à l'âge environ de trois à six mois [2–4], le décès fœtal puis sa calcification. Selon que la calcification intéresse uniquement le fœtus ou les annexes de l'œuf ou les deux, on distingue le lithopédion proprement dit (calcification du fœtus mais pas les annexes), lithokeliphos (les membranes forment une coque calcique alors que le fœtus n'est quasiment pas calcifié) et le lithokeliphopédion (fœtus et annexes sont calcifiés) [5]. Dans notre cas, il s'agit donc d'un lithopédion dont le début remonterait à plus de huit ans. Dans la littérature, deux tiers des patientes avaient un âgesupérieuràquarante ans. Notre patiente avait quarante-six ans ce qui concorde avec les autres études [2–4]. Selon ces auteurs [2–4], la pathologie évoluait depuis quatre à soixante ans. Dans notre cas, le lithokeliphos évoluait depuis environ neuf ans. Un fait important dans notre cas est que la patiente rapporte la notion d'une aménorrhée secondaire de cinq mois associée aux signes sympathiques de la grossesseet ceci quelque temps avant la dernière grossesse normale. Le bas niveau socioéconomique et intellectuel engendre souvent un retard de la première consultation prénatale voir même l'absence total d'un suivi. Ce qui a pour conséquence le non diagnostic de cette grossesse ectopique et son évolution vers le lithopédion. Quant au tableau clinique, il est souvent pauvre voir inexistant [3]. Dans notre cas, la patiente a consulté devant des douleurs pelviennes ainsi que des signes de compression (constipation). Dans cette pathologie, la radiologie est d'un grand apport. Si l'échographie ne fait que diagnostiquer un utérus vide et la présence d'une masse latéro utérine calcifiée, le scanner et surtout l'IRM permettent de faire le diagnostic [2, 4, 6]. Cependant, d'autres diagnostics doivent être éliminés en particuliers les tumeurs ovariennes calcifiées. Dans notre cas, l'échographie a mis en évidence l'utérus vide et une masse antéro utérine calcifiée; quant au scanner, il a confirmé la présence de structures osseuses dans la masse. Quant à la prise en charge, elle reste non codifiée. En effet, si certains auteurs préconisent un traitement chirurgical [7], d'autre, devant la stabilité du tableau, préfèrent l'expectative [2]. Dans tous les cas, la décision de l'ablation chirurgical ou non de la tumeur doit prendre en compte les risques et bénéfices de l'intervention. Dans notre cas, étant donné l'âge de la patiente, l'accessibilité de la masse et les conditions locales qui semblaient favorables, nous avons décidé de pratiquer un geste chirurgical. Celui-ci fut plus compliqué que prévu puisqu'il a nécessité, autre l'ablation du lithopédion, d'une ablation partielle de l'épiploon. Les suites sont simples.

## Conclusion

Le lithopédion est une pathologie rare dont l'installation peut remonter à des dizaines d'années avant le diagnostic. Il peut est de découverte fortuite voire post mortem. Le diagnostic positif est essentiellement radiologique. L'attitude thérapeutique dépend de plusieurs facteurs; elle peut être l'expectative ou plus agressive, chirurgicale.
